# Spatial multiplexed immunofluorescence analysis reveals coordinated cellular networks associated with overall survival in metastatic osteosarcoma

**DOI:** 10.1038/s41413-024-00359-z

**Published:** 2024-09-27

**Authors:** Ryan A. Lacinski, Sebastian A. Dziadowicz, Vincent K. Melemai, Brody Fitzpatrick, John J. Pisquiy, Tanya Heim, Ines Lohse, Karen E. Schoedel, Nicolas J. Llosa, Kurt R. Weiss, Brock A. Lindsey

**Affiliations:** 1 Department of Orthopaedics, West Virginia University School of Medicine, Morgantown, WV 26506 USA; 2 Cancer Institute, West Virginia University School of Medicine, Morgantown, WV 26506 USA; 3 Department of Microbiology, Immunology and Cell Biology, West Virginia University School of Medicine, Morgantown, WV 26506 USA; 4 Bioinformatics Core, West Virginia University School of Medicine, Morgantown, WV 26506 USA; 5 Department of Orthopaedic Surgery, University of Pittsburgh Medical Center, Pittsburgh, PA 15213 USA; 6 Department of Pathology, University of Pittsburgh Medical Center, Pittsburgh, PA 15213 USA; 7 Department of Orthopaedic Surgery, Johns Hopkins University School of Medicine, Baltimore, MD 21287 USA

**Keywords:** Bone cancer, Bone cancer

## Abstract

Patients diagnosed with advanced osteosarcoma, often in the form of lung metastases, have abysmal five-year overall survival rates. The complexity of the osteosarcoma immune tumor microenvironment has been implicated in clinical trial failures of various immunotherapies. The purpose of this exploratory study was to spatially characterize the immune tumor microenvironment of metastatic osteosarcoma lung specimens. Knowledge of the coordinating cellular networks within these tissues could then lead to improved outcomes when utilizing immunotherapy for treatment of this disease. Importantly, various cell types, interactions, and cellular neighborhoods were associated with five-year survival status. Of note, increases in cellular interactions between T lymphocytes, positive for programmed cell death protein 1, and myeloid-derived suppressor cells were observed in the 5-year deceased cohort. Additionally, cellular neighborhood analysis identified an Immune-Cold Parenchyma cellular neighborhood, also associated with worse 5-year survival. Finally, the Osteosarcoma Spatial Score, which approximates effector immune activity in the immune tumor microenvironment through the spatial proximity of immune and tumor cells, was increased within 5-year survivors, suggesting improved effector signaling in this patient cohort. Ultimately, these data represent a robust spatial multiplexed immunofluorescence analysis of the metastatic osteosarcoma immune tumor microenvironment. Various communication networks, and their association with survival, were described. In the future, identification of these networks may suggest the use of specific, combinatory immunotherapeutic strategies for improved anti-tumor immune responses and outcomes in osteosarcoma.

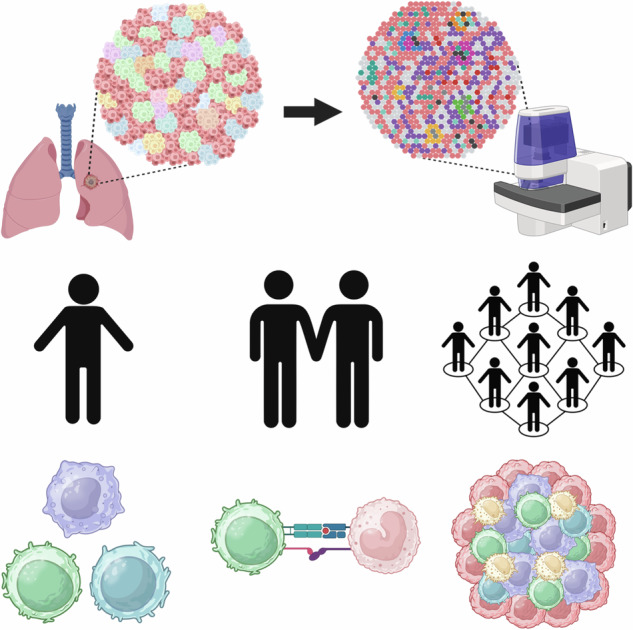

## Introduction

Osteosarcoma (OS) is a tumor of mesenchymal origin and the most common primary bone neoplasm in children and young adults.^1,2^ Primary OS is most often diagnosed in the metaphyseal region of long bones, with gross classification based on tumor location (extra-skeletal, surface, intramedullary).^3,4^ While the presence of malignant cells and deposition of intratumoral osteoid is the basis of histological diagnosis, further classification, based on overall matrix composition, can divide OS tumors into osteoblastic, chondroblastic, and fibroblastic subtypes.^5^ The mainstay standard of care (SOC) therapy for primary malignancy includes surgical resection alongside neoadjuvant and adjuvant multi-drug methotrexate, doxorubicin, and cisplatin (MAP) chemotherapy.^6^ While patients with only localized disease display five-year event free survival rates greater than 75%,^7,8^ those who develop advanced disease, most often in the form of metastases to the lung, have a much worse prognosis. Here, five-year survival decreases to approximately 25%.^9^

Treatment of metastatic disease is predicated on surgical resection of all detectable lesions alongside aggressive, multi-drug chemotherapy.^10^ While the combinatory use of cell cycle-specific and non-specific agents through multi-drug ifosfamide, etoposide, and high-dose methotrexate chemotherapy has shown the greatest tumor control rates, numerous other regimens have also been reported.^11^ Often, patient-specific therapeutic approaches are deployed for management of advanced disease due to a number of underlying patient and tumor-specific factors.^10^ Considering the reduced overall survival rates with advanced disease, clinical trials assessing the efficacy of next-generation targeted therapeutics^12^ and immunotherapies, including checkpoint blockade inhibitors, adoptive cellular therapies, and cancer vaccines,^13,14^ have been conducted. Of note, early results have suggested limited therapeutic efficacy of the immunotherapeutic strategies examined, with failures associated with the complexity and heterogeneity of the OS tumor microenvironment (TME).^15^

The dynamic OS TME, consisting of interactions between vascular, stromal, immune, and tumor cell components within a mineralized extracellular matrix, has, therefore, been the focus of research over the last decade.^7,16–18^ Recent work characterizing the OS TME at single-cell resolution has highlighted the sheer heterogeneity that exists in primary, recurrent, and metastatic OS lesions. These studies have ultimately defined various cellular components and processes associated with therapeutic resistance, metastasis, and overall prognosis.^19–26^ Researchers have also highlighted the presence of various immune escape mechanisms within the OS TME that are associated with poorer patient outcomes and lack of response to immunotherapy.^27^ Recently, Ligon et al. characterized the upregulation of immunomodulatory molecules, including programmed death 1 (PD-1), on tumor infiltrating lymphocytes concentrated at the tumor-lung interface of metastatic lesions. The group also observed the presence of an intratumoral myeloid derived suppressor cell (MDSC) gene signature, associating both phenomena with worse overall survival.^28^

While the single-cell RNA sequencing technique deployed in the above analyses provides the utmost cellular resolution, the organized tissue architecture, which can give direct insight into the cell-to-cell communication networks that may exist within the TME, is unfortunately lost in biospecimen processing. Furthermore, the traditional immunohistochemical and/or immunofluorescent staining techniques are limited by the number of biomarkers that can be simultaneously examined within the same tissue, thereby thwarting in-depth characterization of those cells. As a result, these limitations have been overcome by correlating tissue staining results with additional immunophenotyping (flow cytometry) or genomic (RNA sequencing) analyses. Intercellular communication can then be inferred through gene expression profiling, as evidenced by a recent investigation of known receptor-ligand interactions in Ewing’s and OS.^29^ Fortunately, the ability to achieve single-cell resolution while maintaining tissue architecture is the basis of next-generation spatial omic techniques,^30^ which are used to characterize the metastatic OS immune TME (iTME) in the work presented here.

Recent advancements in spatial, multiplexed immunofluorescence technologies^31^ enable simultaneous classification and phenotypic characterization of cellular populations on formalin-fixed, paraffin-embedded (FFPE) tissues using extensive immunophenotyping panels consisting of DNA-barcoded antibodies. After first demonstration by Goltsev et al.,^32^ this technology, known as co-detection by indexing (CODEX), has been used to characterize the TME of numerous cancer types including, but not limited to, cutaneous T cell lymphoma,^33,34^ colorectal cancer,^35^ glioblastoma,^36^ hepatocellular carcinoma,^37,38^ head and neck squamous cell carcinoma,^39^ melanoma,^40^ pancreatic ductal adenocarcinoma,^41^ as well as follicular lymphoma.^42^ By maintaining the original tissue architecture, various spatial analyses, including calculation of the physical distances between cell types, assessment of direct cellular contacts, as well as determination of multi-cellular, coordinating cellular neighborhoods, can now be conducted.^34,35^ Ultimately, these multiplexed tissue imaging platforms have been proposed to not only provide insight into disease processes, but also transform the field of personalized medicine through the development of next-generation spatial biomarkers of prognosis and treatment response.^43,44^

The purpose of this exploratory study was to spatially characterize the iTME of metastatic OS lung specimens, and the coordinating cellular networks that may exist in these tissues, using the next-generation of CODEX multiplexed immunofluorescence technology, known as PhenoCycler.^45^ To do so, two metastatic OS lung tissue microarrays (TMAs) were imaged with the PhenoCycler Fusion platform [Akoya Biosciences Spatial Tissue Exploration Program (STEP), Marlborough, MA] following staining with oligonucleotide-barcoded PhenoCycler antibodies. Using the Enable Medicine Cloud Platform (Enable Medicine Inc., Menlo Park, CA), we then identified and further characterized the tumor, immune, stromal, and vascular components of the metastatic OS iTME. Importantly, various cell types, cellular interactions, and cellular neighborhoods identified within these specimens were associated with five-year survival status. Our group also proposes the OS Spatial Score, which approximates effector immune activity within these metastatic lesions through the spatial proximity of various immune and tumor cell populations.

## Results

### Specimen selection, patient cohort design, and staining quality assessment

**Fig. 1:**
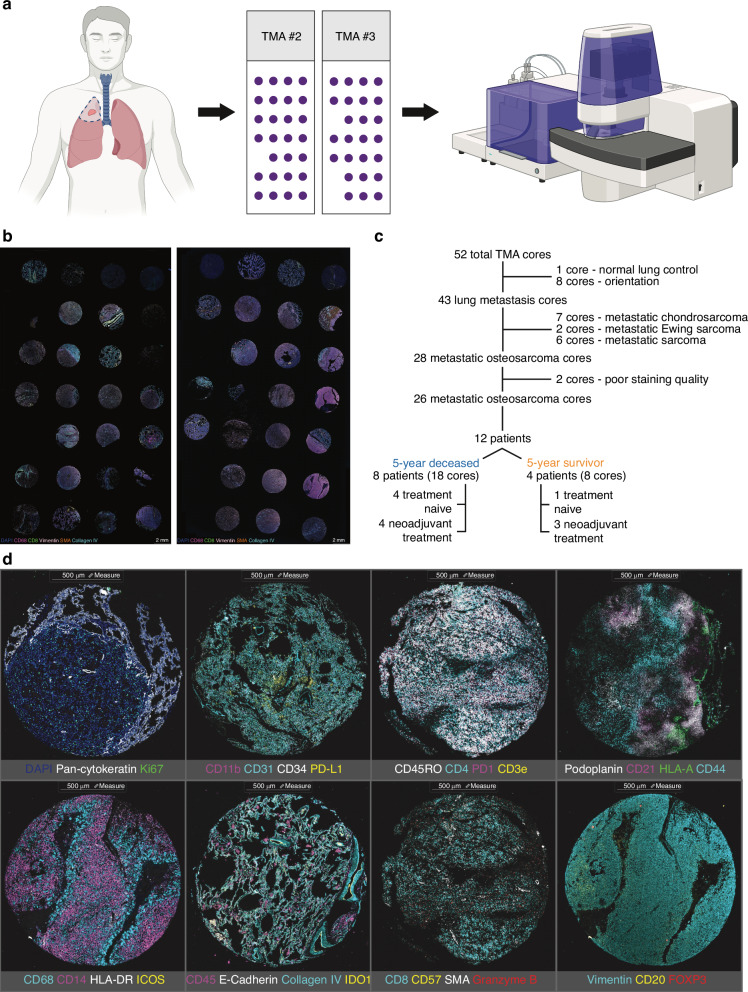
Experimental Design. **a** Overview of experimental design highlighting the use of the PhenoCycler Fusion platform to perform spatial multiplexed immunofluorescence analysis of metastatic osteosarcoma (OS) tissue microarrays (TMAs). Schematic created at BioRender.com. **b** Successful staining and imaging of both metastatic OS TMAs, with DAPI, CD68, CD8, Vimentin, SMA, and Collagen IV staining on whole TMA images presented to highlight successful detection of both structural and immune (myeloid and lymphoid) cell components. **c** Tree diagram depicting the composition of cores from both TMAs and the patient stratification performed for subsequent analysis. **d** Representative images featuring all cellular biomarkers included within downstream analysis following staining quality assessment and control. Scale bar set at 500 µm

Two metastatic OS TMAs were constructed at the University of Pittsburgh Biospecimen Core following analysis of whole-slide Hematoxylin and Eosin (H&E) images and selection of areas of immune infiltration by a board-certified pathologist with expertise in bone and soft-tissue sarcomas. These TMAs were sent to the Akoya Biosciences STEP for staining and subsequent image analysis on the PhenoCycler Fusion platform (Fig. 1a). Whole TMA image analysis indicated successful staining and image acquisition of both metastatic OS TMAs (Fig. 1b). Of the 52 initial cores, two cores were excluded from downstream analysis due to poor staining quality (abnormal DAPI). After removal of orientation/control cores, as well as non-metastatic OS specimens, a total of 26 metastatic OS cores (representative of 12 patients) were selected for downstream analysis. These patients, with ages ranging from 13 to 69, had confirmatory diagnoses of metastatic OS and were treated with various SOC surgery and chemotherapeutic regimens (Table 1). Of note, one patient (included within the 5-year deceased cohort) had a primary tumor classified as a dedifferentiated liposarcoma with divergent (osteosarcomatous) differentiation and definitive bone involvement. Importantly, the metastatic lesion (based on differentiation) was classified as metastatic OS and treated accordingly, ultimately indicating that the aggressive, osteosarcomatous component is what eventually metastasized to the lung. Stratification of these patients based on 5-year survival status identified a 5-year deceased (eight patients, 18 cores in total) and survivor (four patients, 8 cores) cohort for further quantitative analyses. Subsequent stratification of the above cohorts by neoadjuvant chemotherapy exposure (prior to biospecimen collection) was also conducted (Fig. 1c). Staining for each of the 34 biomarkers (Table 2) was then assessed for specificity and signal intensity both internally and by the Akoya Biosciences STEP on experimental, orientation, and control cores. After examination, CD11c and CD163 (non-specific staining), as well as CD-19 and LAG-3 (abnormal staining, lack of signal detection above background on OS cores), were removed from downstream analysis. Adequate specificity and signal intensity was observed for the remaining 30 biomarkers on metastatic OS specimens, as evidenced by the representative staining shown (Fig. 1d).

**Table 1:** Patient demographic and disease information

Patient	Age	Sex	Vital Status	Primary Tumor Site	Metastatic Site(s)	Nodule # and Size (cm)	Metastatic Treatment(s)	Treatment Status at Collection	5-year survival
1	58	M	DWD	LE	RUL	2 (1, 0.5)	(1) Gemcitabine, Paclitaxel (2) Pazopanib (3) Sorafenib	Naïve	D
2	36	M	DWD	UE	RUL	1 (2.5)	(1) Ifosfamide, Etoposide (2) Radiation	Neoadjuvant	D
3	13	M	DWD	LE	Lingula, LLL	UMSB (0.3–1.6)	(1) Cisplatin, Doxorubicin, Methotrexate (2) Ifosfamide, Etoposide, Methotrexate (3) Doxorubicin, Dexrazoxane, Ifosfamide	Neoadjuvant	D
4	24	F	DWD	LE	LLL	4 (0.4–2.2)	(1) Ifosfamide, Etoposide (2) Sorafenib	Neoadjuvant	D
5	69	F	DWD	LE	RUL, RML	2 (0.9, 0.5)	(1) Doxorubicin, Ifosfamide, and Mesna (2) SBRT (3) Liposomal Doxorubicin	Naïve	D
6	56	F	DWD	LE	LLL	4 (1.5–2.5)	Resection only	Naïve	D
7	64	M	DWD	LE	RLL, RUL	UMSB (0.4–1.5)	(1) Ifosfamide, Etoposide (2) Gemcitabine, Docetaxel (3) Liposomal Doxorubicin (4) Pazopanib (5) DMS612	Neoadjuvant	D
8	42	F	DWD	UE	LLL	1 (3.7)	(1) Ifosfamide, Etoposide (2) Gemcitabine, Docetaxel	Naïve	D
9	29	F	A	LE	LLL	1 (0.3)	Cisplatin, Doxorubicin, Methotrexate	Neoadjuvant	S
10	23	F	A	LE	RUL	1 (0.8)	Cisplatin, Doxorubicin, Methotrexate	Neoadjuvant	S
11	25	M	AWD	CF	LLL	UMSB (0.3-6.5)	Resection only	Naïve	S
12	68	M	DWD	LE	LLL	1 (0.9)	Doxorubicin	Neoadjuvant	S

*M* Male, *F* Female, *DWD* Deceased with Disease, *A* Alive, *AWD* Alive with Disease, *LE* Lower Extremity, *UE* Upper Extremity, *CF* Cranio-facial, *RUL* Right Upper Lobe, *LLL* Left Lower Lobe, *RML* Right Middle Lobe, *RLL* Right Lower Lobe, *UMSB* Unmeasurable, *SBRT* Stereotactic body radiation therapy, *D* Deceased, *S* Survivor

**Table 2:** Oligonucleotide-barcoded PhenoCycler antibodies and conditions for TMA staining and analysis

Target	Clone	Vendor	Reporter	Dilution (1:X)	Exposure/ms	Cycle
Major Histocompatibility Complex, class I, A (HLA-A)	EP1395Y	Akoya Biosciences	RX004-AF750	400	150	2
CD34	QBEND/10	Invitrogen	RX025-ATTO550	50	150	2
CD20	L26	Akoya Biosciences	RX007-AF750	50	150	3
CD68	KP1	Akoya Biosciences	RX015-Cy5	500	150	3
Vimentin	O91D3	Akoya Biosciences	RX022-AF750	3 000	150	4
CD8	C8/144B	Akoya Biosciences	RX026-ATTO550	1 000	150	4
CD11c	118/A5	Akoya Biosciences	RX024-Cy5	50	150	4
CD31	EP3095	Akoya Biosciences	RX001-ATTO550	150	150	5
E-Cadherin	4A2C7	Akoya Biosciences	RX014-ATTO550	75	150	5
CD45	D9M81	Akoya Biosciences	RX021-Cy5	400	150	5
Smooth muscle actin (SMA)	1A4	Akoya Biosciences	RX013-AF750	1 600	150	6
CD45RO	UCHL1	Akoya Biosciences	RX017-ATTO550	75	150	6
CD3e	EP449E	Akoya Biosciences	RX045-Cy5	100	150	6
Pan-Cytokeratin	AE1/AE3	Akoya Biosciences	RX019-AF750	1 200	150	7
CD44	156-3C11	Akoya Biosciences	RX005-ATTO550	600	150	7
Major Histocompatibility Complex, class II, DR (HLA-DR)	EPR3692	Akoya Biosciences	RX033-Cy5	200	150	7
GranzymeB (GZMB)	D6E9W	Akoya Biosciences	RX041-ATTO550	25	150	8
CollagenIV	EPR209660	Akoya Biosciences	RX042-Cy5	100	150	8
CD278 (Inducible T-cell costimulatory, ICOS)	D1K2T	Akoya Biosciences	RX054-ATTO550	50	150	9
Forkhead Box P3 (Foxp3)	SP7	Akoya Biosciences	RX031-AF647	50	150	9
Lymphocyte activating 3 (LAG3)	D2G40	CST	RX055-ATTO550	75	150	10
CD163	D6U1J	CST	RX016-AF647	75	150	10
CD19	RM332	RevMab	RX028-ATTO550	75	150	11
CD4	EPR6855	Akoya Biosciences	RX003-Cy5	200	150	11
Marker of proliferation Ki67 (Ki67)	B56	Akoya Biosciences	RX047-ATTO550	100	150	12
CD57	HNK-1	BioLegend	RX020-AF647	75	150	12
CD21	EP3093	Akoya Biosciences	RX032-ATTO550	50	150	13
Programmed death-ligand 1 (PD-L1)	RM320	Akoya Biosciences	RX043-AF647	50	150	13
CD14	EPR3653	Akoya Biosciences	RX037-ATTO550	400	150	14
CD11b	EP1345Y	Abcam	BX030-AF647	100	150	14
Podoplanin (PDPN)	NC-08	Akoya Biosciences	BX023-ATO550	100	150	15
Indoleamine 2,3-dioxygenase-1 (IDO1)	V1NC3IDO	Akoya Biosciences	RX027-ATTO550	100	150	15
Programmed cell death protein 1 (PD-1)	D4W2J	Akoya Biosciences	RX046-AF647	25	150	16

### Unsupervised clustering reveals 45 distinct cellular clusters in OS metastatic lung specimens

Following DeepCell cellular segmentation^46^ and quality control, cellular phenotyping was performed using the Unsupervised Clustering extension of the Enable Medicine Cloud platform. Briefly, unsupervised clustering revealed 45 unique cellular clusters. Clusters were then identified according to hallmark lineage markers and previous publications which identified cellular populations of primary and metastatic OS lesions through single-cell RNA sequencing.^24^ Accordingly, structural, stromal, immune, and tumor cell populations were identified across all metastatic OS cores. Overall, a total of 714 672 cells were segmented and characterized in this analysis (Supplementary File 1). For structural and stromal cells, unsupervised clustering identified Epithelial (Pan-cytokeratin^+^ E-cadherin^+^) and Endothelial (CD31^+^ CD34^+^) cells, Collagen IV^+^ and SMA^+^ Fibroblasts, as well as Lymphatic Endothelial cells [Podoplanin (PDPN)^+^]. For immune cells, unsupervised clustering highlighted various T and B lymphocyte populations and associated subclusters defined by functional markers CD45RO (memory), GranzymeB (GZMB), ICOS, PD-1, IDO1, and/or PDPN. In addition to Natural Killer (NK) cells (defined by CD57 positivity), numerous myeloid lineage cells, including Macrophages (CD68^+^ HLA-DR^+^), Tumor-Associated Macrophages (TAMs, CD68^+^ HLA-DR^-^), Mono-Macs (CD14^+^ CD68^+^), Monocytes (CD14^+^ HLA-DR^+^), Myeloid-Derived Suppressor cells (MDSCs, CD14^+^ HLA-DR^-^), and Neutrophils (CD11b^+^), were also identified.

**Fig. 2:**
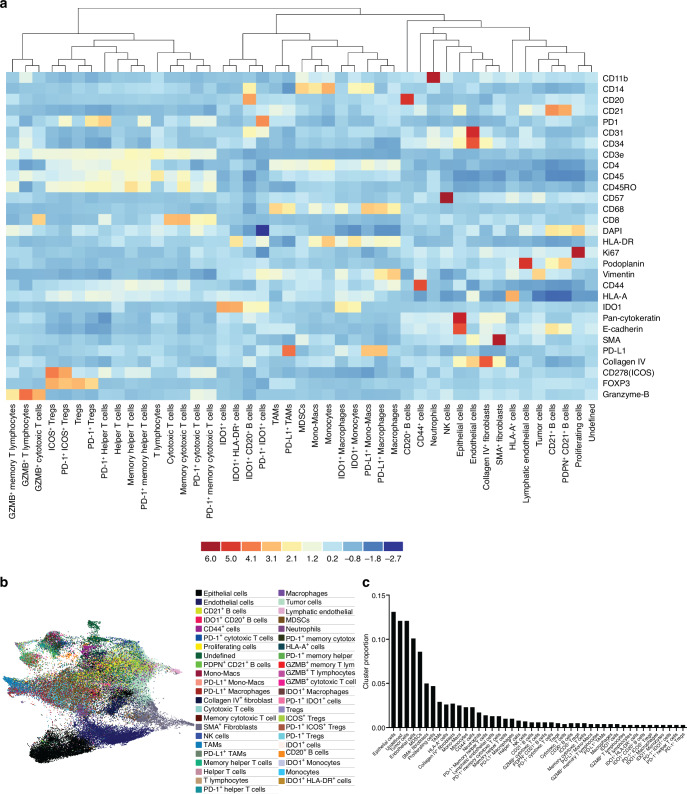
Unsupervised Clustering. **a** Heatmap with row-level Z-score normalization depicting identification of 45 unique cellular clusters and associated phenotypes across all OS metastatic cores examined. **b** UMAP of each cellular cluster from unsupervised clustering. **c** Relative proportion of each cluster across all metastatic OS cores analyzed

Several populations adequately clustered based on staining biomarkers but lacked a defining cellular lineage marker and could not be phenotyped in this analysis. For simplicity, these clusters were defined only by their staining patterns and included IDO1^+^ cells, IDO1^+^ HLA-DR^+^ cells, PD-1^+^ IDO1^+^ cells, CD44^+^ cells, HLA-A^+^ cells, and Ki67^+^ (Proliferating) cells. Finally, both an Undefined (non-cellular artifact and/or population not defined by available biomarkers) and Tumor cell (Vimentin^+^) cluster were also classified during cellular phenotyping (Fig. 2a). Adequate clustering of identified cellular populations was supported by a uniform manifold approximation and projection (UMAP) dimension reduction analysis (Fig. 2b). Epithelial, Tumor, and Endothelial cells were identified as having the greatest relative cluster proportion across all cores. For immune cell populations, myeloid cells predominated, with the greatest cluster proportion evident for MDSCs followed by TAMs, Mono-Macs, and Neutrophils (Fig. 2c). Clustering results were validated for phenotypic accuracy across all OS metastatic cores before further analyses by superimposing each cluster’s expression profile over the identifying cellular overlay within the Visualizer extension of the Enable Medicine Cloud Platform, ensuring robust classification accuracy.

### Cell type analysis reveals distinct immune populations associated with five-year survival

Following cellular phenotyping by unsupervised clustering, various analyses comparing OS patient cohorts (naïve vs. neoadjuvant chemotherapy-treated specimens, 5-year deceased vs. survival cohorts, etc.) were conducted using a Welch’s *t*-test. While this statistical approach is suitable for comparison of smaller, heterogenous datasets (as displayed here), the patients assessed in these analyses were represented on the TMAs often by multiple tissue cores, lending to the use of more stringent mixed models in statistical analyses. Given the exploratory nature of this work, and rarity of metastatic OS disease specimens, the Welch’s *t*-test was chosen for statistical comparisons as it better aligned with our study’s primary objective (identifying and conveying the patterns that may exist in these specimens).

**Fig. 3:**
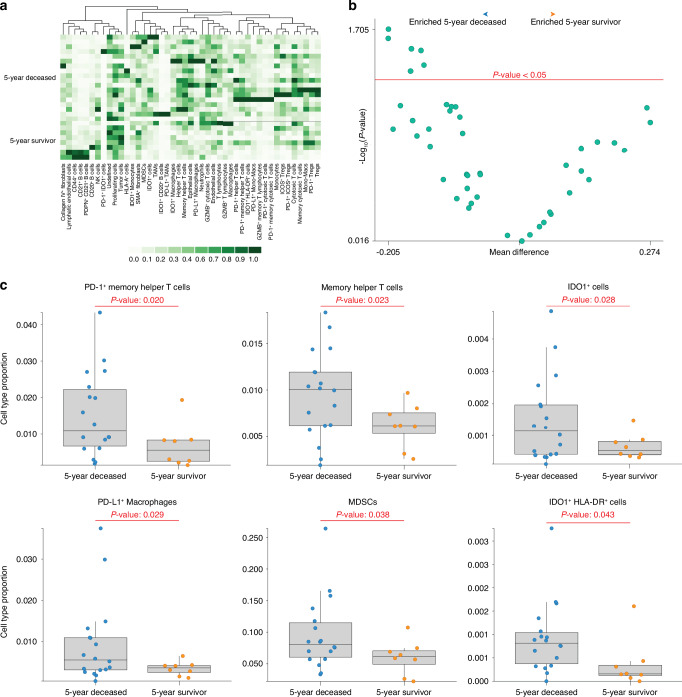
Cell Type Analysis. **a** Heatmap, normalized to Min-Max, depicting the cellular composition of metastatic OS specimens, stratified by 5-year survival status. Each row represents a single core from the TMA. **b** Volcano plot depicting cellular enrichment within metastatic OS specimens, stratified by 5-year survival status. Red line indicates *P* < 0.05, with any cell type above considered statistically significant. **c** Box plots depicting statistically significant cellular population within metastatic OS specimens, stratified by 5-year survival status. Each dot on the box plot denotes a single core from the TMA, with the 5-year deceased cohort representing *n* = 8 patients, 18 cores while the 5-year survivor represents *n* = 4 patients, 8 cores. The upper whisker extends from the upper hinge to the largest value no further than 1.5 times interquartile range (IQR) from the upper hinge, while the lower whisker extends from the lower hinge to the smallest value, at most 1.5 times the IQR from the lower hinge. Welch’s *t*-test was conducted to assess the differences in means between the compared cohorts, with significance set at *P* < 0.05 (Enable Medicine Cloud Platform)

To begin, a cell type analysis comparing naïve and neoadjuvant chemotherapy-treated specimens revealed no significant differences between experimental groups, as the only population enriched (in naïve specimens) was the Undefined cellular cluster (Fig. S1, Supplementary File 2). As a result, patient cohort stratification based on 5-year survival status grouped patients irrespective of treatment exposure prior to sample collection. This finding was unsurprising considering the biased selection of areas of immune infiltration when designing the OS TMAs for our analysis. Differences in cell type proportion between the 5-year deceased (*n* = 8 patients, 18 cores) and survivor (*n* = 4 patients, 8 cores) cohorts were then analyzed (Fig. 3a). As depicted by the volcano plot, distinct cell types were significantly enriched (*P* < 0.05) within the 5-year deceased cohort (Fig. 3b, Supplementary File 3). These populations included PD-1^+^ Memory Helper T cells, Memory Helper T cells, IDO1^+^ cells, PD-L1^+^ Macrophages, MDSCs, as well as IDO1^+^ HLA-DR^+^ cells (Fig. 3c). While not significant, various populations also displayed a trending enrichment in the 5-year deceased cohort including HLA-A^+^ cells (*P* = 0.079), GZMB^+^ Cytotoxic T cells (*P* = 0.080), PD-L1^+^ Mono-Macs (*P* = 0.082), PD-1^+^ Memory Cytotoxic T cells (*P* = 0.082), GZMB^+^ Memory T Lymphocytes (P = 0.088), as well as PD-1^+^ Cytotoxic T cells (*P* = 0.099). Additionally, both PDPN^+^ CD21^+^ B cells (*P* = 0.084) and CD21^+^ B cells (*P* = 0.109) displayed a trending enrichment in the 5-year survivor cohort (Supplementary File 3). This enrichment was particularly driven by what appears to be the presence of early tertiary lymphoid structures (TLS) in two of eight cores (1 patient) within the 5-year survivor cohort.

A subsequent analysis stratifying the neoadjuvant chemotherapy-exposed specimens based on 5-year survival status further supported these findings (Fig. S2a). Importantly, a volcano plot depicted significant enrichment (P < 0.05) of distinct cell types within the 5-year deceased (neoadjuvant) cohort (Fig. S2b, Supplementary File 4). These populations included the previously highlighted IDO1^+^ HLA-DR^+^ cells and PD-1^+^ Memory Helper T cells alongside PD-1^+^ Tregs (Fig. S2C). Similarly, both PD-L1^+^ Mono-Macs (*P* = 0.064) and PD-1^+^ Cytotoxic T cells (*P* = 0.088) again displayed trending enrichment in the 5-year deceased (neoadjuvant) cohort (Supplementary File 4). Overall, these analyses highlight the association between various cellular populations, particularly PD-1 and PD-L1^+^ immune cells, in the metastatic OS TME with 5-year survival status. This relationship appears to be irrespective of treatment exposure status at time of biospecimen collection.

### Cellular interaction analysis details direct cell-to-cell communications associated with five-year survival

**Fig. 4:**
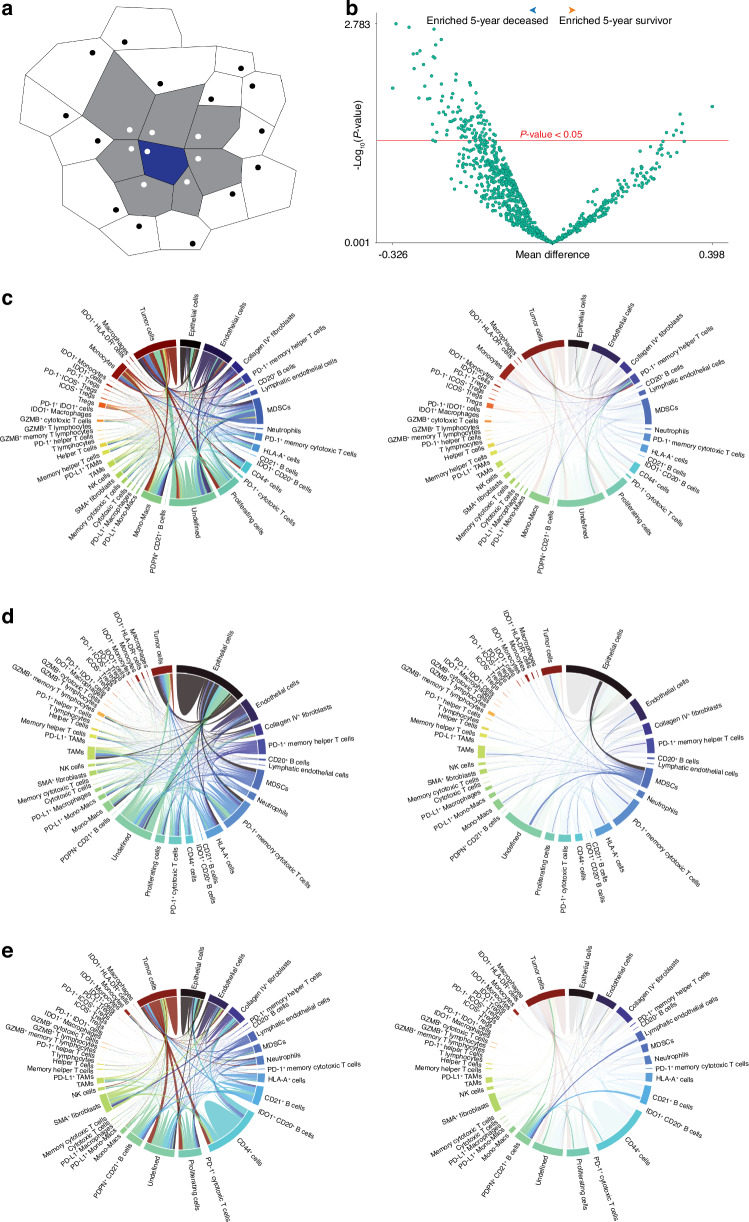
Cellular Interaction Analysis. **a** Schematic detailing how the Delaunay Triangulation method is used to establish cell-to-cell contacts between cellular populations of the iTME. Schematic adapted from the Enable Medicine Inc. User Manual and previous publication.^160^ **b** Volcano plot depicting cellular interactions within metastatic OS specimens, stratified by 5-year survival status. Red line indicates *P* < 0.05, with any cell type above considered statistically significant. **c** Representative interaction chord diagram of a 5-year deceased metastatic OS core (left) with enrichment for PD-1^+^ Memory Helper T cell interactions (right). **d** Representative interaction chord diagram of a 5-year deceased metastatic OS core (left) with enrichment for MDSC interactions (right). **e** Representative interaction chord diagram of a 5-year survivor metastatic OS core (left) with enrichment for PDPN^+^ CD21^+^ B cell interactions (right). The 5-year deceased cohort represents *n* = 8 patients, 18 cores while the 5-year survivor represents *n* = 4 patients, 8 cores. Welch’s *t*-test was conducted to assess the differences in means between the compared cohorts, with significance set at *P* < 0.05 (Enable Medicine Cloud Platform)

Cellular interaction analysis determines the frequency of direct cellular contacts between adjacent cell types (Fig. 4a). These direct cell-to-cell contacts can help to provide insight into the direct cellular communication that may exist within the OS iTME. As depicted by the volcano plot, particular cellular interactions were enriched within the 5-year deceased and survivor cohorts (Fig. 4b, Supplementary File 5). Within the 5-year deceased cohort, there was significant enrichment (*P* < 0.05) of cell-to-cell contact between PD-1^+^ Memory Helper T cells and Tumor cells, Neutrophils, Proliferating cells, CD44^+^ cells, HLA-A^+^ cells, MDSCs, and Macrophages (Fig. 4c). There was also significant enrichment (*P* < 0.05) of cellular interaction between PD-1^+^ Memory Cytotoxic T cells and Tumor cells, Monocytes, and IDO1^+^ cells, among others. Similar interactions were also evident for PD-1^+^ Cytotoxic T cells (Supplementary File 5). Furthermore, the 5-year deceased cohort also featured significant enrichment (*P* < 0.05) of cell-to-cell contacts between MDSCs and IDO1^+^ HLA-DR^+^ cells, PD-1^+^ Cytotoxic T cells, HLA-A^+^ cells, PD-1^+^ Helper T cells, and PD-1^+^ Memory Cytotoxic T cells (Fig. 4d). Analysis also revealed significant enrichment (*P* < 0.05) of interactions between PD-L1^+^ myeloid lineage cells and Tregs, Helper T cells, and NK cells within this same cohort (Supplementary File 5). Ultimately, these data highlight what appears to be direct cellular interaction between both tumor and immunosuppressive myeloid-lineage cells, often positive for PD-L1 or IDO1, with infiltrating lymphoid populations, often positive for PD-1, within the 5-year deceased cohort.

Within the 5-year survivor cohort, there was significant (*P* < 0.05) enrichment of cellular interaction between PDPN^+^ CD21^+^ B cells, CD21^+^ B cells, Proliferating cells, as well as Lymphatic Endothelial cells with Tumor cells (Fig. 4e). These interactions ultimately highlight the likely formation of tumor-communicating early TLSs within 2 cores (1 patient) of the survivor cohort. Overall, these data indicate that distinct cell-to-cell interactions occur within the OS iTME when comparing 5-year deceased and survivor cohorts. Similar, yet less prominent, interactions were also evident following additional stratification by neoadjuvant chemotherapy exposure status, particularly for both PD-1^+^ Memory Helper and PD-1^+^ Memory Cytotoxic T cell populations (Fig. S3, File 6).

### Cellular neighborhood analysis highlights conserved, coordinated networks within the OS iTME

**Fig. 5:**
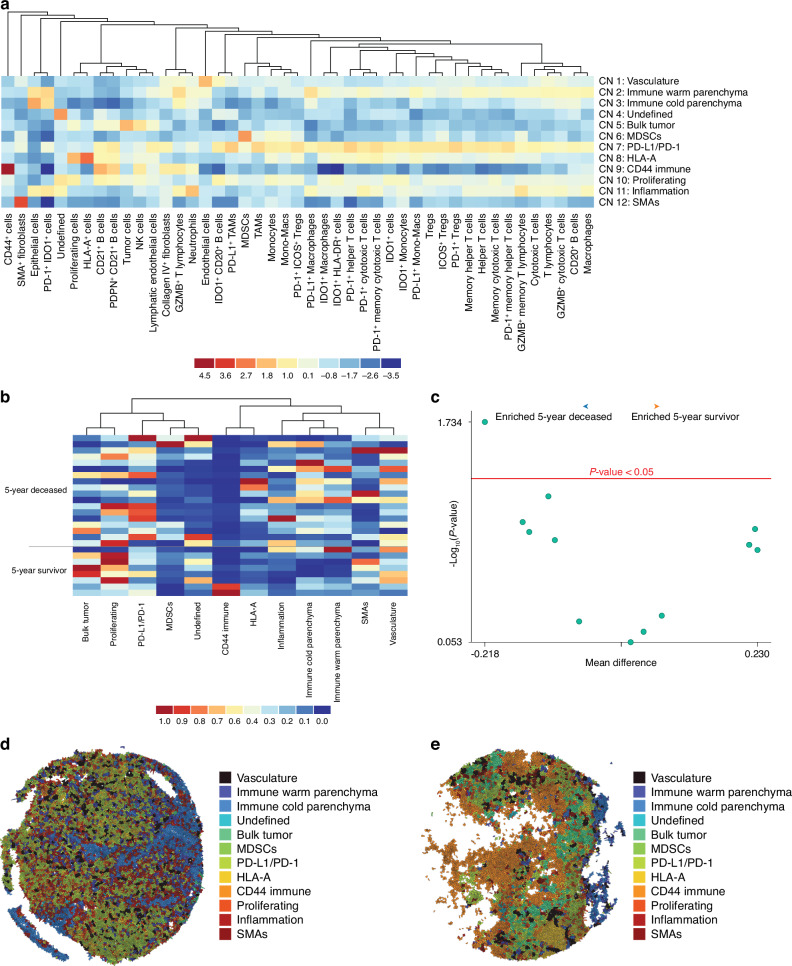
Cellular Neighborhood Analysis. **a** Cellular neighborhood (CN) identification in all metastatic OS specimens using k-means clustering, 10 neighbors, and 12 CN classifications. Rows represent the CNs while columns represent cell type. Heatmap is scaled by rows (0-1, giving cell type proportions within each neighborhood) and then by columns (global abundance of cell types) followed by a log_2_ transformation. **b** Heatmap, normalized to Min-Max, depicting the CN composition of metastatic OS specimens, stratified by 5-year survival status. Each row represents a single core from the TMA. **c** Volcano plot depicting CN enrichment within metastatic OS specimens, stratified by 5-year survival status. Red line indicates *P* < 0.05, with any CN above considered statistically significant. **d** Representative Voronoi plot of a 5-year deceased metastatic OS core. **e** Representative Voronoi plot of a 5-year survivor metastatic OS core. The 5-year deceased cohort represents *n* = 8 patients, 18 cores while the 5-year survivor represents *n* = 4 patients, 8 cores. Welch’s *t*-test was conducted to assess the differences in means between the compared cohorts, with significance set at *P* < 0.05 (Enable Medicine Cloud Platform)

Cellular neighborhood (CN) classification helps identify conserved, spatially organized, multi-cellular communication networks within the iTME that may correlate with treatment response and/or overall survival. Using k-means clustering, 12 conserved CNs were classified across all metastatic OS cores. These CNs were defined based on their overall cellular composition and classified as Vasculature, Immune Warm and Cold Parenchyma, Bulk Tumor, MDSCs, PD-L1/PD-1, HLA-A, CD44 Immune, Proliferating, Inflammation, SMAs, and Undefined CNs (Fig. 5a). The differences in CN proportion between the 5-year deceased (*n* = 8 patients, 18 cores) and survivor (*n* = 4 patients, 8 cores) cohorts were then analyzed (Fig. 5b). As depicted by the volcano plot, the Immune Cold Parenchyma CN was significantly enriched (*P* < 0.05) within the 5-year deceased cohort (Fig. 5c, Supplementary File 7). This finding supports nearly a decade of experimental data which suggests that a lack of immune infiltration is associated with worse overall survival in OS patients.^9^ Additionally, a trending enrichment of the MDSC CN (*P* = 0.068) was evident, as depicted by a representative 5-year deceased Voronoi plot (Fig. 5d).

While only trending, both the Inflammation CN, composed predominantly of Neutrophils, as well as the PD-L1/PD-1 CN, highlighting immune checkpoint receptor/ligand interaction, were also enriched within the 5-year deceased cohort (Supplementary File 7). Furthermore, the Bulk Tumor, Proliferating, and CD44 Immune CNs, which particularly compose the TLSs evident within 2 cores (1 patient) of the 5-year survival cohort, are enriched albeit not significantly (Fig. 5e, Supplementary File 7). A subsequent CN analysis stratifying the neoadjuvant chemotherapy-exposed specimens based on 5-year survival status showed similar yet non-significant findings (Fig. S4, Supplementary File 8). Overall, these results indicate that both a lack of effector immune infiltration, as indicated within the Immune Cold Parenchyma CN, coupled with increased MDSC infiltration and communication, as indicated by the MDSC CN, are associated with poorer overall survival.

### OS Spatial Score associates intratumoral effector immune status with long term survival

**Fig. 6:**
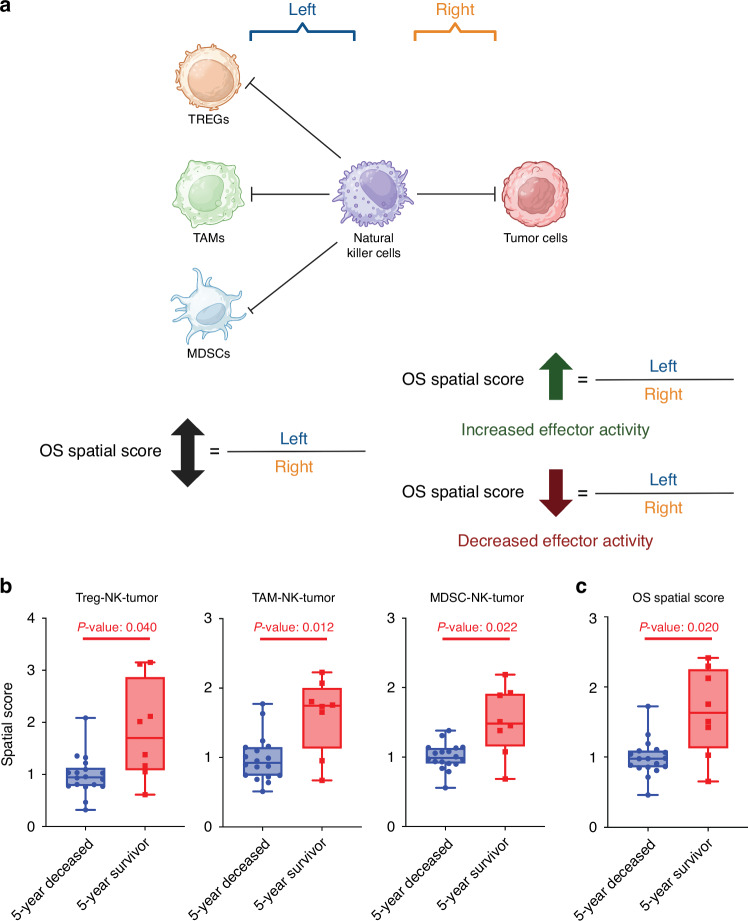
OS Spatial Score. **a** Schematic diagram depicting the calculation of the OS Spatial Score by dividing the distance of Tregs, TAMs, and MDSCs to Natural Killer (NK) cells (left) by the distance of Tumor cells to NK cells (right). Increased effector function correlates with an increased Spatial Score, while decreased effector function correlates with a decreased Spatial Score. Schematic created at BioRender.com. **b** Treg-, TAM-, and MDSC-NK-Tumor Spatial Scores for metastatic OS specimens, stratified by 5-year outcome. Each dot on the box plot denotes a single core from the TMA. **c** Composite OS Spatial Score depicts increased effector activity in the 5-year survivor cohort. Each dot on the box plot denotes a single core from the TMA, with whiskers extending from the Min to Max value. The 5-year deceased cohort represents *n* = 8 patients, 18 cores while the 5-year survivor represents *n* = 4 patients, 8 cores. Welch’s *t*-test was conducted to assess the differences in means between the compared cohorts, with significance set at *P* < 0.05 (GraphPad Prism 10)

Previous literature suggests that Tregs, TAMs, and MDSCs are the critical cellular populations responsible for coordinating an immunosuppressive network within the TME.^47^ Additionally, it is well understood that NK cell infiltration and activation are critical for effector, anti-tumor immune responses in OS.^48–55^ Our cell type analysis indicated that while numerous immunosuppressive cell populations were enriched within the 5-year deceased cohort, NK cells were enriched within the 5-year survivor cohort (Supplementary File 3). It was then hypothesized that the physical proximity of NK cells to Tregs, TAMs, MDSCs, and Tumor cells could approximate overall effector immune activity within OS metastatic lung specimens, and this effector immune activity would then associate with survival. Similar to previous work,^34^ the OS Spatial Score was calculated by dividing the median distance from these immunosuppressive cell populations to NK cells (left) by the median distance between Tumor cells to NK cells (right). A greater Spatial Score, indicative of increased distance between immunosuppressive and effector immune cells and/or decreased distance between effector immune cells and tumor cells, indicates superior effector-immune activity. Conversely, a lower Spatial Score, indicative of decreased distance between immunosuppressive and effector immune cells and/or increased distance between effector immune cells and tumor cells, indicates inferior effector-immune activity (Fig. 6a). Significant increases (*P* < 0.05) in Treg-, TAM-, and MDSC-NK-Tumor Spatial Scores were evident in the 5-year survivor cohort (Fig. 6b, Supplementary File 9). As a result, a significant increase (*P* < 0.05) in the composite OS Spatial Score was also observed in this group (Fig. 6c, Supplementary File 9). Ultimately, these data indicate that an increased OS Spatial Score, or increased effector immune activity, as determined by the physical distance of NK cells to both immunosuppressive immune and tumor cells populations, correlates with better overall survival.

## Discussion

Various PD-1^+^ and PD-L1^+^ immune cell populations were enriched within the 5-year deceased cohort including, among others, PD-1^+^ Memory Helper T cells. Importantly, PD-1 expression on peripheral Cytotoxic and Helper T cells previously correlated with OS metastatic disease progression.^56^ Investigators have also reported that infiltrating Cytotoxic T cells in metastatic OS lesions displayed a dysfunctional, exhausted phenotype hallmarked by increased PD-1 expression.^57,58^ This finding was further confirmed by Ligon et al. who described an increase in tumor infiltrating lymphocytes positive for PD-1 and PD-L1, among other immunoregulatory molecules, within OS lung metastases.^28^ Considering PD-1/PD-L1 overexpression within OS is associated with higher mortality risk,^59^ numerous clinical trials assessing checkpoint blockade immunotherapy against PD-1, PD-L1, among other immunoregulatory molecules (alone or in combination), have been conducted. Unfortunately, results have suggested limited therapeutic efficacy of immunotherapies amongst the OS patients trialed.^60–64^

Numerous mechanisms for immune checkpoint inhibitor failures have been proposed, with other components of the iTME implicated in the observed immune escape by OS tumor cells.^65^ Of these contributing components, our 5-year deceased cohort exhibited significant increases in MDSCs, with enrichment of additional immunosuppressive myeloid-lineage populations (ex. PD-L1^+^ Macrophages, PD-L1^+^ Mono-Macs). Considering the well documented role of MDSCs in cancer progression,^66,67^ therapeutic targeting of these cells has been suggested to improve patient outcomes.^68^ More recently, MDSCs have been directly implicated in resistance to immune checkpoint blockade therapy by heightening immunosuppressive signals within the TME.^69–72^

Within OS, previous literature has suggested that MDSCs are significantly elevated within patient peripheral blood and are capable of inhibiting T cell activation. Additionally, recruitment of MDSCs to the OS iTME ultimately resulted in an immunosuppressive network capable of reducing the efficacy of anti-PD-1 immunotherapy. Checkpoint blockade efficacy, however, was enhanced with the addition of a PI3Kδ/γ inhibitor, responsible for impeding MDSC-mediated immunosuppression and enabling both enhanced immune infiltration and innate immune activation in preclinical studies.^73^ Other research has suggested that MDSCs increase their infiltration into the OS TME through the SDF-1/CXCR4 axis, where they are then able to inhibit Cytotoxic T cell expansion to reduce anti-PD-1 therapeutic efficacy. In support of this theory, the addition of AMD3100, a CXCR4 antagonist, alongside anti-PD-1 immunotherapy proved synergistic in a murine model of OS.^74^ Furthermore, IL-18 has been shown to correlate with MDSC levels in both OS patient peripheral blood and tumors. Preclinical delivery of anti-IL-18 therapy, alongside anti-PD-1, decreased MDSCs, increased T lymphocyte infiltration, and reduced overall tumor burden.^75^

Ultimately, our results support previous literature’s findings which suggest that infiltrating MDSCs and PD-1^+^ T lymphocytes in the OS TME are associated with poorer overall survival. This work, to our knowledge, is the first to directly associate the frequency of cellular interactions between MDSCs and PD-1^+^ T lymphocytes, including PD-1^+^ Cytotoxic T cells, PD-1^+^ Helper T cells, PD-1^+^ Memory Cytotoxic T cells, and PD-1^+^ Memory Helper T cells, with 5-year survival status. This work is also the first, to our knowledge, to associate an MDSC-driven, infiltrating myeloid CN with poorer overall survival, highlighting the development of a coordinated, immunosuppressive signaling network within these tissues. Overall, these findings highlight the importance of MDSC-T lymphocyte interactions in this patient population. These results also provide further support for a likely mechanism of MDSC-mediated resistance to immune checkpoint blockade therapy in previous OS clinical trials. Here, an increase in cell-to-cell interactions between MDSCs and PD-1^+^ T lymphocytes, and coordinated immunosuppressive signaling thereafter, likely subverts any alleviation provided by antibody blockade of the PD-1/PD-L1 axis. While further analysis of immunotherapy-treated specimens with this platform is required to support this theory, efforts to target MDSCs alongside this immunoregulatory axis are likely necessary for potent anti-tumor immune responses in metastatic OS. In the future, spatial multiplexed immunophenotyping to identify these cells and their interactions may suggest combinatory MDSC-PD-1/PD-L1 checkpoint blockade regimens, as opposed to SOC therapy, in subsets of OS patients.

Furthermore, cell type analysis highlighted a significant increase in two unidentified IDO1^+^ cellular clusters within the 5-year deceased cohort. Cellular interactions between both PD-1^+^ Memory Cytotoxic T cells and PD-1^+^ Cytotoxic T cells with various IDO1^+^ populations, including IDO1^+^ cells (both), IDO1^+^ Monocytes (both), and IDO1^+^ Macrophages (PD-1^+^ Cytotoxic T cells only), were also enriched in this cohort. Importantly, increased IDO1 expression is well regarded as a biomarker of poorer overall survival across many solid tumors.^76,77^ Researchers have suggested IDO1 as a target of next generation immunotherapies, ultimately capable of alleviating IDO-induced immunosuppression in the TME.^78–81^ Regarding OS, Ligon et al. noted an increase in IDO1 expression within metastatic OS lesions, particularly at the tumor-lung interface.^28^ In accordance with the data reported here, investigators have associated IDO1 expression with OS disease progression and worsening overall prognosis, particularly through suppression of effector immune cells.^82^

Our spatial multiplexed analysis highlights direct cellular interactions between IDO1^+^ clusters and infiltrating T lymphocytes, proposing the use of IDO inhibitors alongside checkpoint blockade immunotherapy for certain OS patient subsets. The efficacy of IDO inhibitors for treatment of OS has been assessed in both preclinical and clinical settings. Interestingly, therapeutic efficacy of a nano-hyaluronic acid metal-organic framework for combinatory chemo-immunotherapy of OS was associated with both a reduction in MDSCs and inhibition of IDO activity.^83^ Bi et al. also reported therapeutic efficacy of combinatory IDO therapy in a murine model of OS, with the IDO inhibitor NLG919 enhancing cytotoxic T cell activity.^84^ Furthermore, combinatory inhibition of transforming growth factor-beta receptor type 1 (TGFβR1) and IDO1 through galunisertib and epacadostat, respectively, showed preliminary promise in a murine model of OS when combined with cytotoxic chemotherapy.^85^ Unfortunately, early results from a phase II clinical trial assessing pembrolizumab (anti-PD-1) in combination with the IDO1 inhibitor, epacadostat, for treatment of advanced sarcoma suggested the regimen was associated with limited anti-tumor responses.^86^ With most data suggesting that IDO inhibitors are only synergistic in a combinatory immunotherapy setting,^87^ further research is needed to determine the most efficacious regimens while evaluating potential biomarkers for candidate stratification in this patient population.

Our analysis also highlighted the presence of 12 conserved CNs throughout OS metastatic lesions. Of those, the Immune-Cold Parenchyma CN was determined to be significantly increased within the 5-year deceased cohort. While our group preferentially selected areas of immune infiltration (as determined by H&E) for the curation of our TMAs, lack of robust immune infiltration within metastatic lesions was still associated with poorer 5-year survival. Numerous prognostic gene signatures which correlate tumor immune infiltration with treatment response and survival have been described, with a lack of immune infiltration correlating with poorer patient outcomes.^88–92^ Previous work by Ligon et al. also reported the concentration of tumor infiltrating lymphocytes at the tumor-lung interface, as opposed to the metastasis interior, was associated with worse overall survival.^28^ Furthermore, mRNA expression profiling of infiltrating immune cells in OS tissues highlighted the presence of four distinct patient clusters, of which three were considered immune “cold” and a singular cluster was considered immune “hot”. Unsurprisingly, patients within the immune “hot” cluster reported better overall outcomes.^93^ Even the most recent single-cell RNA sequencing studies have identified a lower risk OS patient cohort with increased immune cell infiltration.^25^ Overall, our CN analysis supports nearly a decade of previously published literature which correlates poor immune infiltration with worsening overall survival in OS patients.

While only trending, our spatial analysis also described the presence of tertiary lymphoid-like structures within 2 cores (1 patient) of the 5-year survivor cohort. Intratumoral TLSs have been investigated in numerous cancer types.^94–98^ These structures ultimately resemble those found in secondary lymphoid organs and contain a B-cell rich follicle surrounded by a T cell rich zone and neighboring dendritic cells (DCs). In addition to a reticular fibroblast network, TLSs are characterized by high endothelial venules (HEVs) that allow infiltration of immune cells. It is then antigen presentation by surrounding DCs which generates effector memory cytotoxic and helper T cells, alongside antibody-producing plasma cells, for enhanced anti-tumor immune responses.^94,99–102^ Importantly, TLS formation is now well regarded as a biomarker of prolonged survival and response to immunotherapy in numerous cancer types.^103–114^ In an advanced sarcoma clinical trial, a subset of TLS-positive soft-tissue sarcoma patients demonstrated significantly greater overall response rates to immunotherapy when compared to an all comers cohort.^115,116^ To our knowledge, there are limited data to suggest that these structures form in primary or metastatic OS lesions.

Our group describes these observed structures as “early” TLSs^114^ due to the presence of a B-cell rich follicle, defined here as a collection of PDPN^+^ CD21^+^ B cells, CD21^+^ B cells, and Lymphatic Endothelial cells, but lacking T cell rich zone. Interestingly, we also describe a surrounding CD44^+^ stromal network which appears to support TLS formation in both cores. CD44 is a plasma membrane glycoprotein that binds to hyaluronan (hyaluronic acid – HA) of the extracellular matrix and, as a cancer stem-cell marker,^117^ has been previously associated in OS with higher tumor grade,^118^ metastasis,^119^ and chemo-resistance.^120–122^ Our data suggest that these CD44^+^ stromal cells (of mesenchymal, fibroblast orgin^123^) likely play a role in TLS development by enhancing leukocyte infiltration and then coordinating subsequent B-cell rich follicle and T cell rich zone organization. In support of this theory, it is well known that immunofibroblasts/stromal cells are essential for the organization of TLSs in various tissues, including within tumors.^124–126^ Previous literature also suggests that lymphocytes, migrating through secondary lymphoid tissues, likely use CD44 to bind HA on stromal cells for aggregation and structure formation.^127^ Additionally, CD44 has been shown to play a role in B cell lymphopoiesis,^128^ likely supporting the formation of a B-cell rich follicle in these tissues. Furthermore, a strong correlation between CD44 positivity and helper T cell infiltration has been reported in OS.^129^ Of note, cytotoxic and helper T cells of a formed TLS also shift to an effector memory phenotype highlighted by increased CD44 expression,^130^ further supporting CD44’s role in these potent inflammatory processes.^131^ To our knowledge, these data are the first to characterize the presence of a CD44^+^ stromal network associated with the formation of TLSs in metastatic OS specimens. While more research in OS is necessary, these data suggest that TLSs are also associated with better overall survival in this patient population.

Finally, this work is the first to describe the OS Spatial Score, which uses the median distance between immunosuppressive immune and tumor cells to NK Cells to approximate effector immune activity within metastatic OS lesions. Considering that NK cell crosstalk with other TME components is known to modulate their activity,^132,133^ our group hypothesized that a spatial biomarker to estimate their effector function would correlate with 5-year survival status. As highlighted previously, literature suggests that MDSCs,^134,135^ Tregs,^136,137^ and TAMs^138^ are responsible for inhibiting NK cell activity through increased immunosuppressive signaling within the TME.^47^ NK cell infiltration within the OS iTME is critical for anti-tumor immune responses and is ultimately associated with better long term survival.^48–55^ Methods to treat OS using NK-based immunotherapies are well underway,^139–144^ including efforts to activate NK cell activity through IL-15 stimulation.^145,146^ Of note, Cruz et al. reported that the spatial localization of NK cells near MHC class I was prognostic in soft-tissue sarcoma,^147^ opening the possibility for the development of a NK-based spatial biomarker in this malignancy. Importantly, the OS Spatial Score was significantly increased in 5-year survivors, ultimately suggesting an increase in NK cell effector immune activity within this cohort. While the OS Spatial Score has shown preliminary promise, this measurement only postulates effector immune function based on the physical distance between various immune and tumor cells. Direct analysis of cellular signaling (through expression profiling) or ex vivo functional assessment is necessary to confirm distance-specific effector differences between our patient cohorts.

While we indicate that the OS Spatial Score could likely be used as a spatial biomarker of overall survival in this disease, more importantly, we believe this measure could help dictate the type of immunotherapy a patient receives in future clinical practice. As an example, if a patient were to present with a low OS Spatial Score or lacking effector activity of NK cells, NK-cell activation therapies (such as intranasal IL-15^145^) could be deployed. These next-generation therapies could then activate residing NK cells or drive infiltration of newly activated NK cells to increase the effector NK-Tumor interactions within the TME (and therefore the overall Spatial Score) for better anti-tumor immune responses and patient outcomes. While a patient presenting with a high OS Spatial Score endogenously attained a spatial organization favoring effector NK activity, immune checkpoint inhibitors could likely help ensure sustained responses by preventing downregulatory signals by tumor or regulatory immune cells in the TME. Whether the OS Spatial Score could be utilized to dictate the type of immunotherapy patients would receive in future human clinical practice ultimately requires further validation.

While novel in our analyses, there are a few inherent limitations to the study presented here. First, due to non-specific staining of both CD163 and CD11c, M2-like Macrophages and Dendritic cell populations, respectively, were not identified within our unsupervised clustering analysis. Before further experimentation using this platform, the selection and validation of different antibody clones, with re-examination of staining patterns on both normal and diseased tissues, will be required. Furthermore, considering that the first step in the analysis pipeline involves automated segmentation of cells based on their nuclei and/or plasma membrane, any multi-nucleated cells are segmented (by their nuclei) into multiple individual cells. In our study, multi-nucleated osteoclasts and/or giant cells were apparent within some OS specimens on visual inspection. Considering an osteoclast-specific biomarker^148^ was not included within our experimental panel, these multi-nucleated cells, positive for macrophage lineage markers,^149,150^ were segmented into individual cells and then included within various macrophage/TAM clusters according to their staining patterns. Use of an osteoclast-specific biomarker will be necessary to more accurately phenotype these cells in future analyses.

Tumor cells were identified by positivity for the common mesenchymal marker, Vimentin.^151^ While Vimentin positivity on OS and other tumors of mesenchymal origin is well known,^152^ this biomarker is also present on the surface of other mesenchymal components of the TME.^153^ Therefore, it is likely that various non-cancerous mesenchymal stromal cells, also positive for Vimentin, were included within our Tumor cell cluster. Future analyses may utilize additional biomarkers such as KPNA2,^154^ Erzin,^155,156^ among many others,^157^ to better phenotype cancerous OS cells. The use of additional biomarkers for mesenchymal stromal cells of interest, such as cancer associated fibroblasts,^158^ may also prove useful. Unfortunately, separating OS tumor cells from these mesenchymal counterparts will still likely prove difficult due to their similar staining patterns.^153^

Due to the scarcity of these rare metastatic OS specimens, a limited number of patients (12 patients, 26 cores) were analyzed in this study. As a result, both naïve and neoadjuvant chemotherapy-exposed specimens were grouped for downstream analyses into their respective 5-year survival cohorts. While there were no statistical differences in relative cluster proportion between naïve and neoadjuvant chemotherapy-exposed specimens, various cell populations were indeed associated with treatment status. Importantly, similar findings were apparent when analyzing only the neoadjuvant chemotherapy-exposed specimens, stratified by 5-year survival status, providing support to the general findings from the grouped cohort. Collection of additional patient specimens, with expansion of both the naïve and neoadjuvant cohorts, will be the basis of future experimentation. Moreover, given the exploratory nature of our study, limited sample size, and known heterogeneity of the OS TME, the Welch’s *t*-test was used for statistical comparison between various patient cohorts. While a Welch’s *t*-test is suitable for comparisons of smaller, heterogenous sample groups as analyzed here, most patients assessed in these analyses were represented on the OS TMA by multiple tissue cores (a repeated measure). We acknowledge that considering these samples as independent assessments is an inherent limitation in our analyses. However, in the context of our experimental design (exploratory and descriptive in nature), aimed at generating hypotheses and identifying patterns within these heterogenous OS metastases, we avoided the use of more stringent mixed models for statistical comparisons to prevent overlooking potentially important phenomena within these specimens. Future confirmatory studies which further investigate the spatial organization of the metastatic OS iTME and the communication networks that may exist will deploy more rigorous statistical assessments alongside power analyses to guide expansion of our patient cohorts and better avoid possible type I or II error. Additionally, Kaplan–Meier survival plots were constructed for the previously highlighted cell types, cellular neighborhoods, as well as the novel OS Spatial Score following cohort stratification into high and low groups based on median values. While similar trends emerged in these analyses, none of the corresponding survival curves were considered statistically significant by Log-rank (Mantel-Cox) test. Again, this finding is more than likely related to the limited number of patients included within these analyses and needed expansion of our patient cohort to further support these findings (Fig. S5).

In conclusion, this exploratory work presents an in-depth spatial, multiplexed immunofluorescence analysis of metastatic OS lung specimens using the Akoya Biosciences PhenoCycler Fusion platform. Through phenotypic characterization by unsupervised clustering, and subsequent stratification by five-year outcomes and neoadjuvant chemotherapy status, various cell types, interactions, and CNs were found to be associated with five-year survival status. Furthermore, our group presents the OS Spatial Score, which approximates effector immune activity within OS metastatic lesions. This next-generation spatial biomarker of effector immune activity was significantly increased within the 5-year survivor cohort, directly associating the spatial proximity of various immune and tumor cell populations with survival. Future work will expand our patient cohorts to further support these experimental findings. Additional analyses of immunotherapy-treated metastatic OS specimens will also be conducted to provide evidence for the hypotheses proposed here. These data may also support the use of combinatory immunotherapeutic strategies in various OS patient subsets, particularly after stratification by spatial TME analysis. The data displayed here also highlight the importance of inducing immune cell infiltration and, possibly, the formation of TLSs^159^ in the OS tumor microenvironment for more positive patient outcomes. In addition to helping recognize possible candidates for immunotherapeutic treatment, these data demonstrate that therapeutic efforts to improve immune cell penetration through this often dense and complex tumor stroma could likely improve patient survival. Ultimately, this work lays the foundation for further spatial characterization of metastatic OS tumors while initiating the development of a prognostic, spatial biomarker of long term survival. These data will also invigorate the development of new immunotherapeutic strategies, targeting distinct cellular communication networks that exist in the iTME, for improved patient outcomes in this disease.

Supplementary information accompanies the manuscript on the Bone Research website http://www.nature.com/boneres.

## Materials and methods

### Patients, tissue preparation, processing, and TMA construction

The human subject research protocol and subject informed consent were reviewed and approved by the University of Pittsburgh Medical Center (UPMC) Institutional Review Board (IRB STUDY 19060152). Written informed consent was obtained from all participants (Table 1) before study entry, according to guidelines. Formalin-fixed paraffin-embedded (FFPE) material from 13 cases of pulmonary OS metastases from 2010–2021 were obtained from the UPMC Department of Pathology. One case was subjected to decalcification by formic acid. Hematoxylin and Eosin (H&E) slides were reviewed by a senior musculoskeletal pathologist (KS) and regions of immune infiltration were selected for tissue microarray (TMA) construction. The TMA was constructed utilizing 2 mm core punches on a Beecher MTA-1 manual arrayer instrument (Beecher Instruments, Inc, Prairie, WI), with sectioning and placement onto standard poly-l-lysine-coated microscope slides according to the Akoya Biosciences PhenoCycler Fusion User Guide, Sample Preparation Guidelines for FFPE samples.

### Tissue staining and image acquisition

FFPE human metastatic OS TMAs were analyzed using the PhenoCycler Fusion platform of the Spatial Tissue Exploration Program (STEP, Akoya Biosciences). Briefly, FFPE TMAs were dewaxed and rehydrated following standard histology methods. Epitope retrieval was performed using Tris-EDTA pH9 for 20 min in a programmable pressure cooker (Instant Pot^TM^). After allowing the pressure cooker to cool and depressurize naturally, the TMAs were bleached by immersion in a solution of 4.5% (w/v) hydrogen peroxide (H_2_O_2_) and 20 mmol/L sodium hydroxide (NaOH) in phosphate buffered saline (PBS) under bright white LED light (A4-sized, Aibecy A4 Ultra Bright 25’000 Lux LED Light Box-Tracing Pads). The TMAs were then stained with a mixture of oligonucleotide-barcoded PhenoCycler antibodies, post-fixed according to the user manual, and imaged on the PhenoCycler Fusion platform. Prior to imaging, a reporter plate with up to three complementary oligonucleotide-fluorophore reporters per cycle was prepared according to the user manual. The antibodies and conditions used for biomarker detection and analysis [in addition to 4’,6-diamidino-2-phenylindole (DAPI) – nuclear] are found in Table 2. Staining was then assessed for specificity and signal intensity both internally and by the Akoya Bioscience STEP.

### Cellular segmentation

All downstream analysis was performed using the Enable Medicine Cloud Platform (Enable Medicine Inc.) according to their standard analysis workflow. Cellular segmentation was performed using the DeepCell segmentation^46^ extension of the Enable Medicine Cloud Platform according to user guidelines. Briefly, for nuclear segmentation, the DeepCell deep learning model for image segmentation (version 0.12.6) was used with image microns per pixel = 0.51, tile size = 16 384, segmentation type = nuclear, nuclear biomarker=DAPI, and cell area threshold = 400 µm^2^. Nuclear segmentation was followed by whole cell segmentation and biomarker expression generation with tile size = 16 384, number of dilations = 9, segmentation method = dilation, and cell area threshold = 400 µm^2^. Cell segmentation results were then validated for their accuracy using the Visualizer application of the Enable Medicine Cloud Platform and the whole cell visual overlay.

### Cellular quality control

Cellular quality control was performed using the Cell Quality Control extension of the Enable Medicine Cloud platform according to user guidelines. Briefly, after cellular segmentation, poor quality cells were removed before downstream analysis using four metrics including cellular size (abnormally small or large), DNA signal (mean pixel intensity from DNA channel), signal sum (total raw signal intensity across all channels, with poorly stained areas having a low signal sum and artifacts tending to have abnormally high signal sum), as well as signal coefficient of variation (vector of biomarker intensities for each segmented cell). Cells that passed quality control were included within all downstream analyses.

### Cellular phenotyping: unsupervised clustering

Cellular phenotyping was performed using the Unsupervised Clustering extension of the Enable Medicine Cloud platform according to user guidelines. Briefly, Leiden clustering with a resolution = 1 was performed following biomarker expression normalization using the following biomarkers: CD11b, CD14, CD19, CD20, CD21, CD31, CD3e, CD4, CD45, CD57, CD68, CD8, Collagen IV, E-Cadherin, FOXP3, Pan-cytokeratin, Podoplanin, SMA, and Vimentin. Here, a Uniform Manifold Approximation and Projection (UMAP) is generated using the scanpy package (Python) through the scanpy.pp.neighbors function to generate neighbors, with n_neighbors = 30. The scanpy.tl.umap function is used to generate the UMAP plot with min_dist argument = 0.000 1. Subclustering analysis was then performed on each identified parent cluster according to user guidelines. Here, variable clustering biomarkers were used according to the population of interest with n_neighbors = 50, 30, or 10, min_dist = 0.000 1, and clustering resolution = 0.5 or 0.1. Cellular clusters were annotated using the generated UMAP colored by cluster, UMAP colored by marker expression, heatmap of the median marker expression, image overlays of clusters and their key marker expression, as well as cluster statistics (relative proportion per core). Unsupervised clustering and sub-clustering results were validated using the Visualizer application of the Enable Medicine Cloud Platform. Here, the identified cluster and sub-cluster’s visual overlay was validated for each characterizing marker’s immunofluorescence to ensure classification accuracy. Markers CD11c and CD163 (non-specific staining), as well as CD-19 and LAG-3 (abnormal staining, lack of signal detection above background on OS cores), were removed from all downstream analyses. Cell types (frequency) were then analyzed within the Explorer application of the Enable Medicine Cloud platform.

### Spatial analysis: cellular interactions

Cellular interactions were calculated within the Explorer application of the Enable Medicine Cloud platform using the Delaunay Triangulation method according to user guidelines. Voronoi diagrams were generated using the d3-delaunay package (JavaScript), with filtering of cells above the cell area threshold (400 µm^2^) and cells with internal angles < 45 degrees. Cell-cell interactions were then calculated based on the frequency of direct cellular contacts between cell types by counting the total number of interactions observed divided by the total number of interactions within an individual core.

### Spatial analysis: cellular neighborhoods

Cellular neighborhoods were defined using the k-nearest-neighbors approach within the Cell Neighborhood extension of the Enable Medicine Cloud platform according to user guidelines and previous publication.^35^ Here, the cell types of every annotated cell’s k-nearest spatial neighbor (with k = 10) in Euclidean space were identified. A k-means clustering approach was then used to determine repeated cell neighborhoods across the dataset by grouping the local neighborhoods into a predefined number of clusters (neighborhoods = 12) based on the mean composition of cellular phenotypes.

### Spatial analysis: neighbor distance and Spatial Score

The proximity of two cell types was calculated using the Spatial Neighbor Distance extension of the Enable Medicine Cloud platform according to user guidelines. Here, the Euclidean distance from the cell centroid of cell type 1 to the cell centroid of cell type 2 was calculated. Cell centroids represent the cell’s location in the tissue X/Y coordinates as determined by the dilated cell segmentation mask. Using the median neighbor distance, the Spatial Score was calculated. The spatial relationship between immunosuppressive immune cells (Tregs, TAMs, and MDSCs), effector immune cells (NK cells), and tumor cells was used to approximate overall effector-immune activity within the OS metastatic lesions. To calculate the Spatial Score, the median distance from an immunosuppressive Treg, TAM, or MDSC to NK cell (left) was divided by the median distance between Tumor cells to NK cells (right) for each OS metastatic core. The OS Spatial Score was calculated by averaging the individual Spatial Scores from Treg-, TAM-, and MDSC-NK-Tumor interactions.

### Kaplan–Meier survival plot construction and analysis

Kaplan–Meier (KM) survival plots were constructed using GraphPad Prism 10 (GraphPad Software, Boston, MA) according to the GraphPad Statistics Guide. Briefly, average proportions for various cell types and cellular neighborhoods as well as the average OS Spatial Score were calculated for all twelve patients included within previous analyses by averaging all values from each patient’s representative cores. For each respective measure, patients were then stratified into high (HIGH) and low (LOW) groups based on the calculated median value. Censoring of subjects occurred according to survival status as of last known follow up (April 2023), with censored subjects indicated by plot symbols on each survival curve. Comparison of survival curves between high and low groups was conducted using the Log-rank (Mantel-Cox) test, with *P*-value < 0.05 considered statistically significant.

### Data visualizations and statistical analysis

Data visualizations were generated using both the Enable Medicine Cloud Platform and GraphPad Prism 10 software. Briefly, Voronoi diagrams were generated using the Enable Medicine Cloud Platform Explorer application and the d3-delaunay package (JavaScript). For optimization, cells larger than 400 µm^2^ and cells with internal angles measuring <45 degrees were filtered out. Interaction chord diagrams were generated using the Enable Medicine Cloud Platform Explorer application and the d3-chord package (JavaScript). Each cell type is represented by a segment of the overall circle, with the size of each segment proportional to the cell type’s frequency. Segments were connected by ribbons which represent cell-cell interaction of two cell types. The thickness of the ribbon represents the frequency of this cell-cell interaction. Heatmaps were generated using the Enable Medicine Cloud Platform Explorer application and the d3-heatmap package (JavaScript). Furthermore, volcano plots were generated using the Enable Medicine Cloud Platform. Here, the x-axis represents the log-fold change (LFC) in feature abundance between the compared groups, while the y-axis represents the negative logarithm (base 10) of the *P*-value [-log_10_(*P*-value)]. For each volcano plot, a Welch’s *t*-test was used to compare the means of the compared cohorts. Given the exploratory nature of this study, the inherent heterogeneity evident in the OS TME, and the limited number of patient samples assessed, each core of the TMA was evaluated as an independent assessment. The choice of using the Welch’s *t*-test is particularly informed due to its suitability for smaller, heterogenous sample cohorts, non-reliance on equal variance between groups, and alignment with the exploratory and descriptive nature of our study. Cohort box plots were generated using the Enable Medicine Cloud Platform Explorer application. The upper whisker extends from the upper hinge to the largest value no further than 1.5 times interquartile range (IQR) from the upper hinge, while the lower whisker extends from the lower hinge to the smallest value, at most 1.5 times the IQR from the lower hinge. Each dot on the box plot is representative of an individual core on the OS TMAs. Finally, for Spatial Score analysis, the 5-year deceased cohort was compared to the 5-year survivor cohort using a Welch’s *t*-test (GraphPad Prism 10).

## Supplementary information

Supplementary Figures and Legends

Supplementary File 1

Supplementary File 2

Supplementary File 3

Supplementary File 4

Supplementary File 5

Supplementary File 6

Supplementary File 7

Supplementary File 8

Supplementary File 9

## Data Availability

Data reported within this manuscript are available in supporting supplementary files or upon reasonable request to the corresponding author.
