# Supplementary material for: Spatial multiplexed immunofluorescence analysis reveals coordinated cellular networks associated with overall survival in metastatic osteosarcoma
Source: Bone Res. 2024 Sep 27;12:55. doi: 10.1038/s41413-024-00359-z (PMC11436896; doi:10.1038/s41413-024-00359-z)

**Supplementary information**

**Supplementary File 1.xlsx – Unsupervised Clustering, Cell Count:** Results from unsupervised clustering including both cluster proportion and cell count for all OS metastatic specimens analyzed.

**Supplementary File 2.xlsx – Cell Type, Naïve vs. Neoadjuvant:** Associated statistical data from Volcano plot comparing the cell types between naïve and neoadjuvant chemotherapy exposed patients.

**Supplementary File 3.xlsx – Cell Type, 5-year deceased vs. survivor:** Associated statistical data from Volcano plot comparing the cell types between 5-year deceased and survivor cohorts.

**Supplementary File 4.xlsx – Cell Type, 5-year deceased (neoadjuvant) vs. survivor (neoadjuvant):** Associated statistical data from Volcano plot comparing the cell types between 5-year deceased (neoadjuvant) and survivor (neoadjuvant) cohorts.

**Supplementary File 5.xlsx – Cellular Interaction, 5-year deceased vs. survivor:** Associated statistical data from Volcano plot comparing the cellular interactions between 5-year deceased and survivor cohorts.

**Supplementary File 6.xlsx – Cellular Interaction, 5-year deceased (neoadjuvant) vs. survivor (neoadjuvant):** Associated statistical data from Volcano plot comparing the cellular interactions between 5-year deceased (neoadjuvant) and survivor (neoadjuvant) cohorts.

**Supplementary File 7.xlsx – Cellular Neighborhood, 5-year deceased vs. survivor:** Associated statistical data from Volcano plot comparing the cellular neighborhoods between 5-year deceased and survivor cohorts.

**Supplementary File 8.xlsx – Cellular Neighborhood, 5-year deceased (neoadjuvant) vs. survivor (neoadjuvant):** Associated statistical data from Volcano plot comparing the cellular neighborhoods between 5-year deceased (neoadjuvant) and survivor (neoadjuvant) cohorts.

**Supplementary File 9.xlsx – OS Spatial Score:** Median measured distances between various cell populations used for calculation of Spatial Scores.

**Supplementary Figure 1, related to Figure 2: Naïve vs. Neoadjuvant. (A)** Heatmap, normalized to Min-Max, depicting the cellular composition of metastatic OS specimens, stratified by treatment status at time of sample collection. Each row representing a single core from the TMA. **(B)** Volcano plot depicting cellular enrichment within metastatic OS specimens, stratified by treatment status at time of sample collection. The naïve cohort represents n = 5 patients, 13 cores while the neoadjuvant cohort represents n = 7 patients, 13 cores. Welch’s t-test was conducted to assess the differences in means between the compared cohorts, with significance set at P < 0.05 (Enable Medicine Cloud Platform).

**
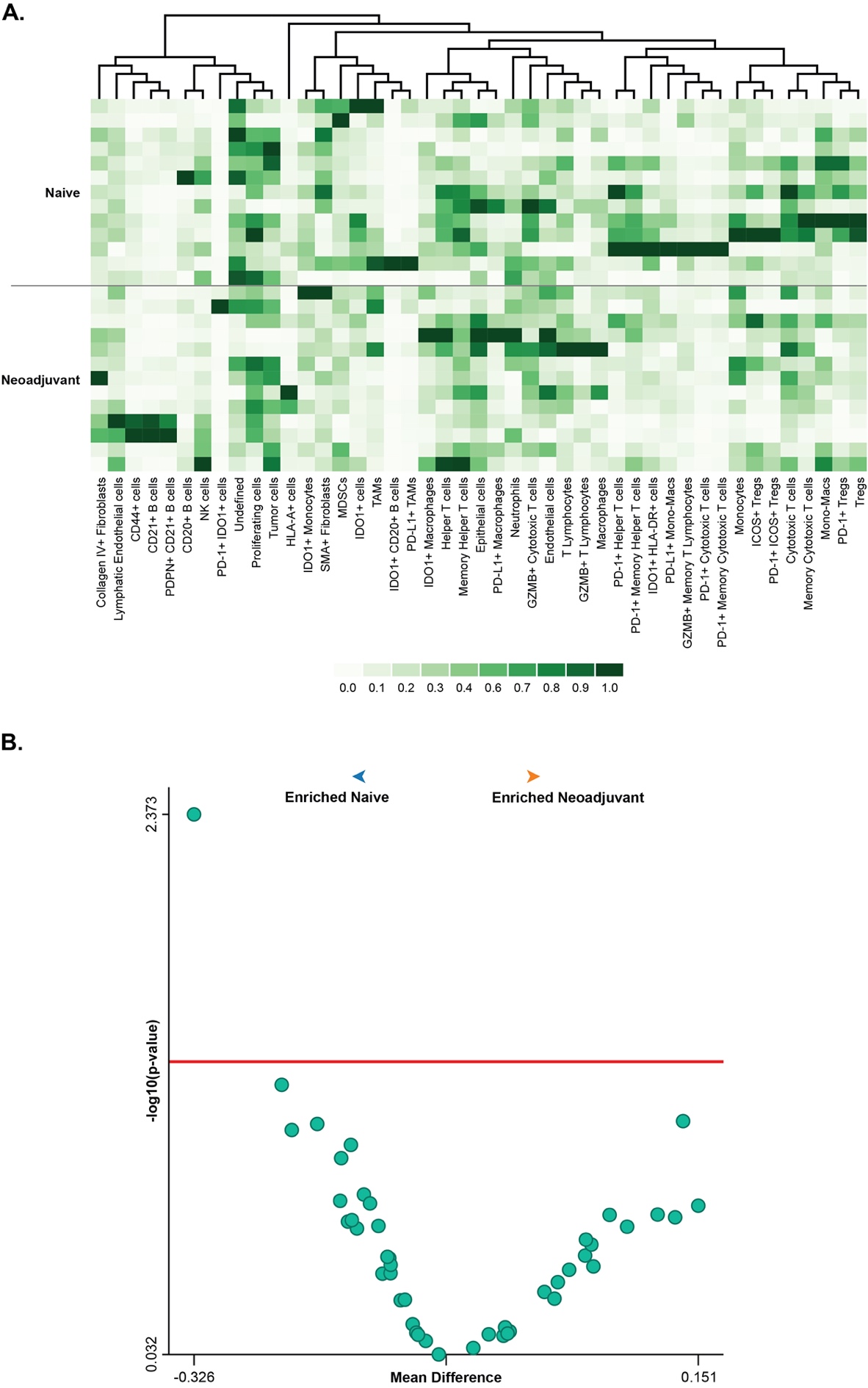
**

**Supplementary Figure 2, related to Figure 3: Neoadjuvant Cell Type Analysis. (A)** Heatmap, normalized to Min-Max, depicting the cellular composition of neoadjuvant-metastatic OS specimens, stratified by 5-year survival status. Each row represents a single core from the TMA. **(B)** Volcano plot depicting cellular enrichment within neoadjuvant-metastatic OS specimens, stratified by 5-year survival status. Red line indicates P < 0.05, with any cell type above considered statistically significant. **(C)** Box plots depicting statistically significant cellular populations within neoadjuvant-metastatic OS specimens, stratified by 5-year survival status. Each dot on the box plot denotes a single core from the TMA, with the 5-year deceased (neoadjuvant) cohort representing n = 4 patients, 9 cores and the 5-year survivor (neoadjuvant) representing n = 3 patients, 4 cores. The upper whisker extends from the upper hinge to the largest value no further than 1.5 times interquartile range (IQR) from the upper hinge, while the lower whisker extends from the lower hinge to the smallest value, at most 1.5 times the IQR from the lower hinge. Welch’s t-test was conducted to assess the differences in means between the compared cohorts, with significance set at P < 0.05 (Enable Medicine Cloud Platform).

**
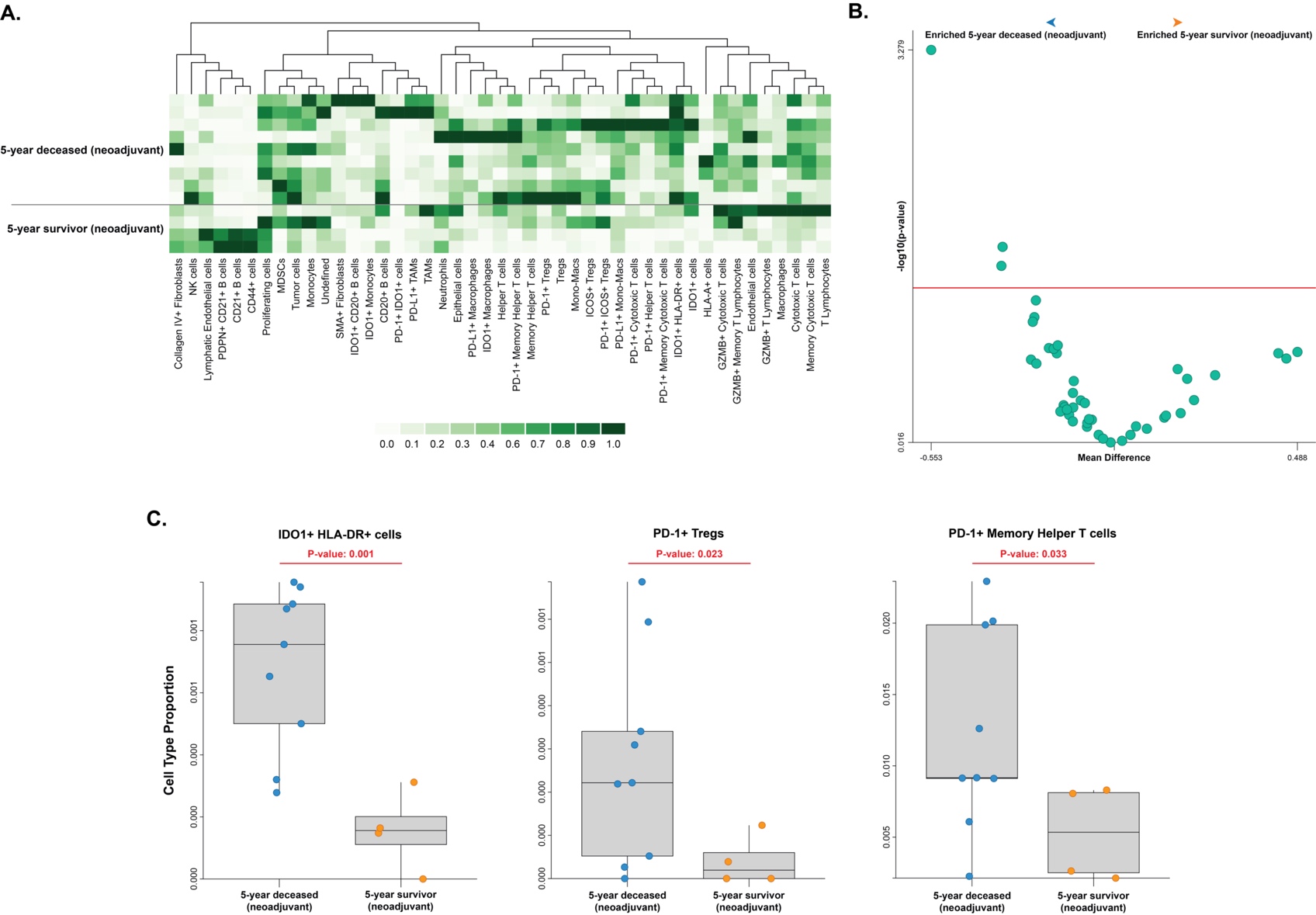
**

**Supplementary Figure 3, related to Figure 4: Neoadjuvant Cellular Interaction Analysis. (A)** Volcano plot depicting cellular neighborhood enrichment within neoadjuvant-metastatic OS specimens, stratified by 5-year survival status. Red line indicates P < 0.05, with any cell neighborhood above considered statistically significant. **(B)** Representative interaction chord diagram of a 5-year deceased, neoadjuvant-metastatic OS core (left) with enrichment for PD-1+ Memory Helper T cell (right) and PD-1+ Memory Cytotoxic T cell (bottom) interactions. The 5-year deceased (neoadjuvant) cohort represents n = 4 patients, 9 cores while the 5-year survivor (neoadjuvant) represents n = 3 patients, 4 cores. Welch’s t-test was conducted to assess the differences in means between the compared cohorts, with significance set at P < 0.05 (Enable Medicine Cloud Platform).

**
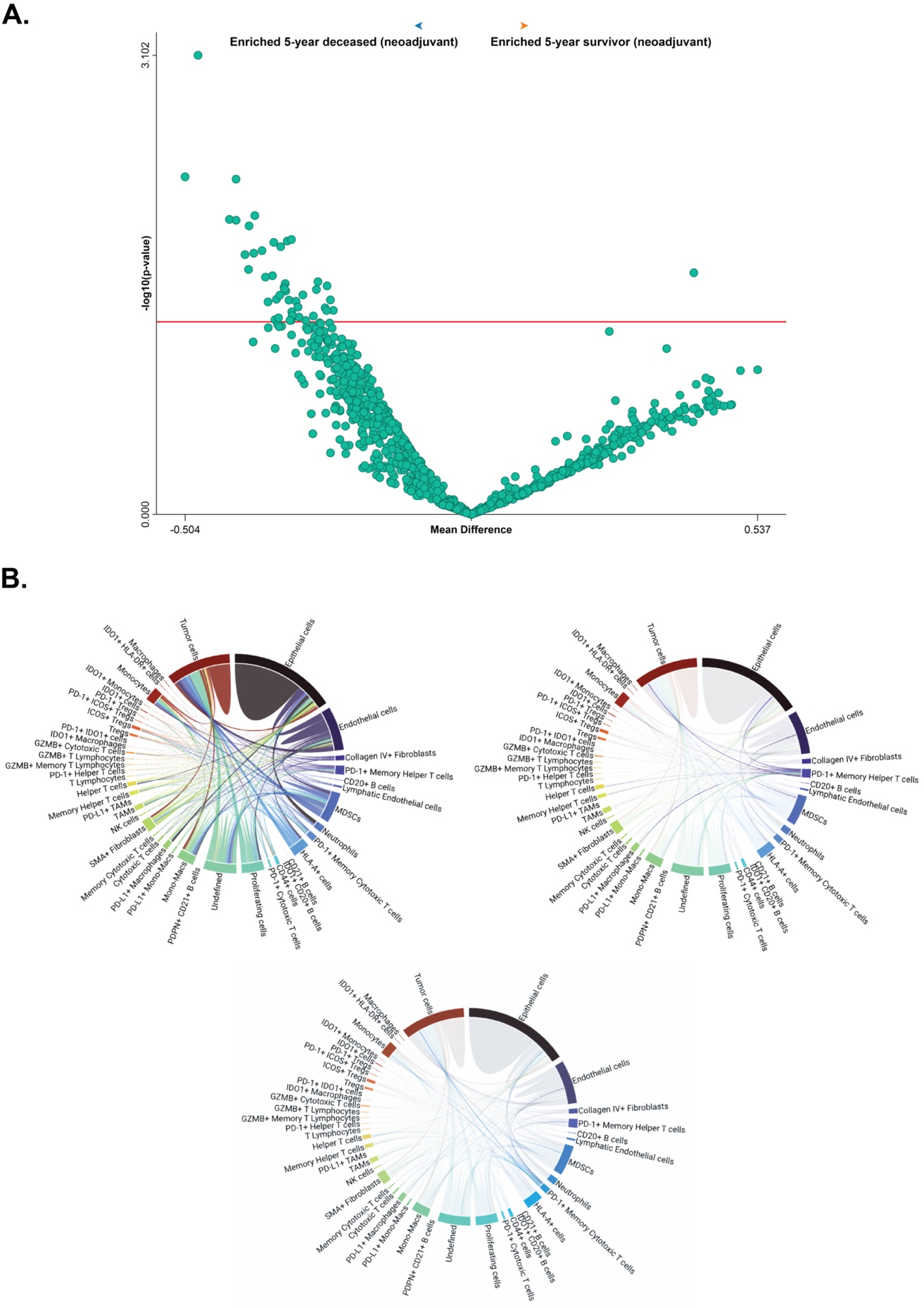
**

**Supplementary Figure 4, related to Figure 5: Neoadjuvant Cellular Neighborhood Analysis. (A)** Heatmap, normalized to Min-Max, depicting the cellular neighborhood composition of neoadjuvant-metastatic OS specimens, stratified by 5-year survival status. Each row represents a single core from the TMA. **(B)** Volcano plot depicting cellular neighborhood enrichment within neoadjuvant-metastatic OS specimens, stratified by 5-year survival status. Red line indicates P < 0.05, with any cell neighborhood above considered statistically significant. The 5-year deceased (neoadjuvant) cohort represents n = 4 patients, 9 cores while the 5-year survivor (neoadjuvant) represents n = 3 patients, 4 cores. Welch’s t-test was conducted to assess the differences in means between the compared cohorts, with significance set at P < 0.05 (Enable Medicine Cloud Platform).


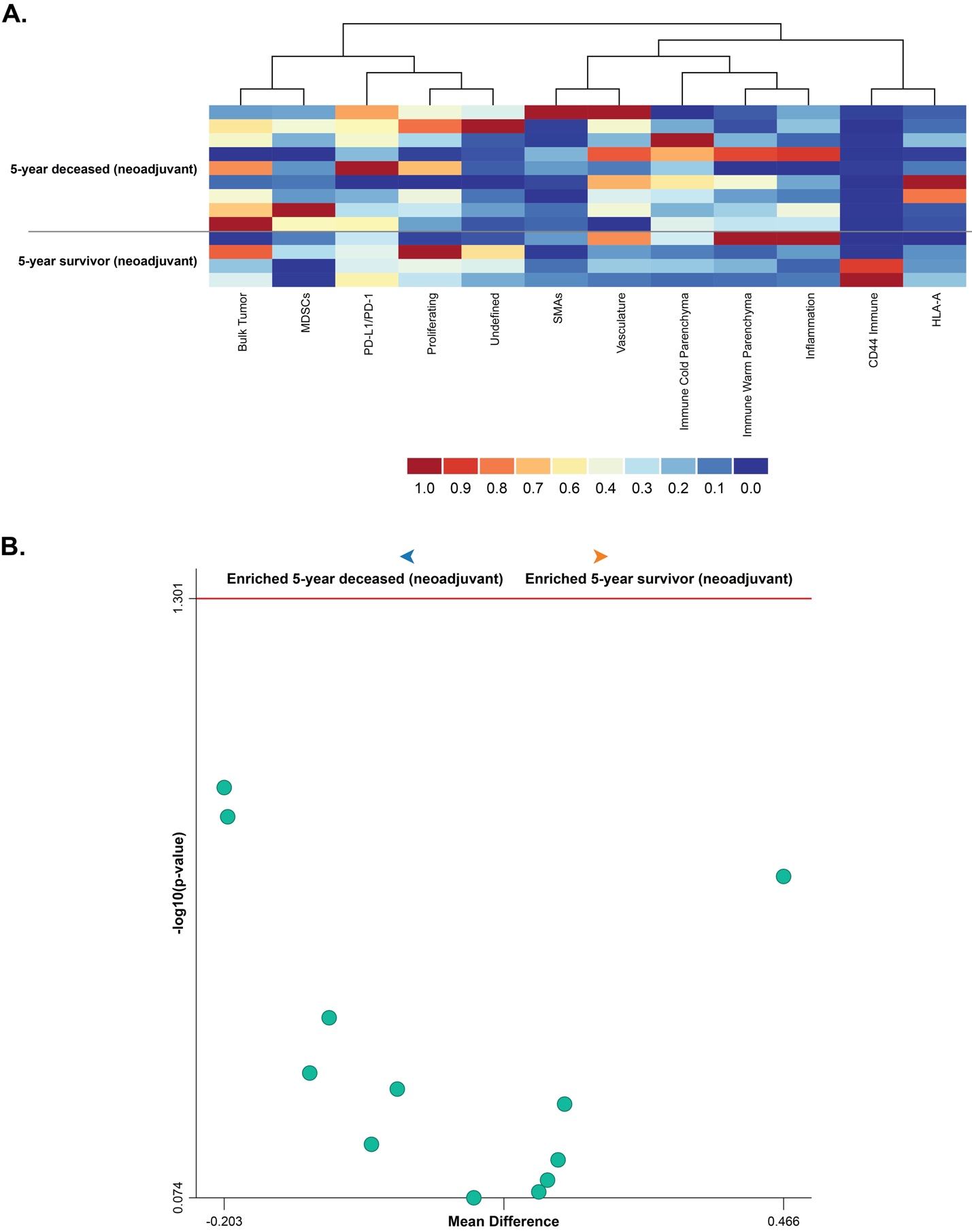


**Supplementary Figure 5: Kaplan Meier Survival Curve Analysis.** Kaplan-Meier survival plots stratifying all metastatic OS patients (n = 12) by median relative cluster proportion for PD-1+ Memory Helper T cells **(A)**, Memory Helper T cells **(B)**, IDO1+ cells **(C)**, PD-L1+ Macrophages **(D)**, MDSCs **(E)**, and IDO1+ HLA-DR+ cells **(F)**, median neighborhood cell proportion for the Immune Cold Parenchyma CN **(G)**, PD-1/PD-L1 CN **(H)**, and MDSC CN **(I**), as well as median OS Spatial Score **(J)**. Survival curve comparison conducted using the Log-rank (Mantel-Cox) test, with the corresponding P-value indicated for each comparison. Plot markers indicate censored events only.


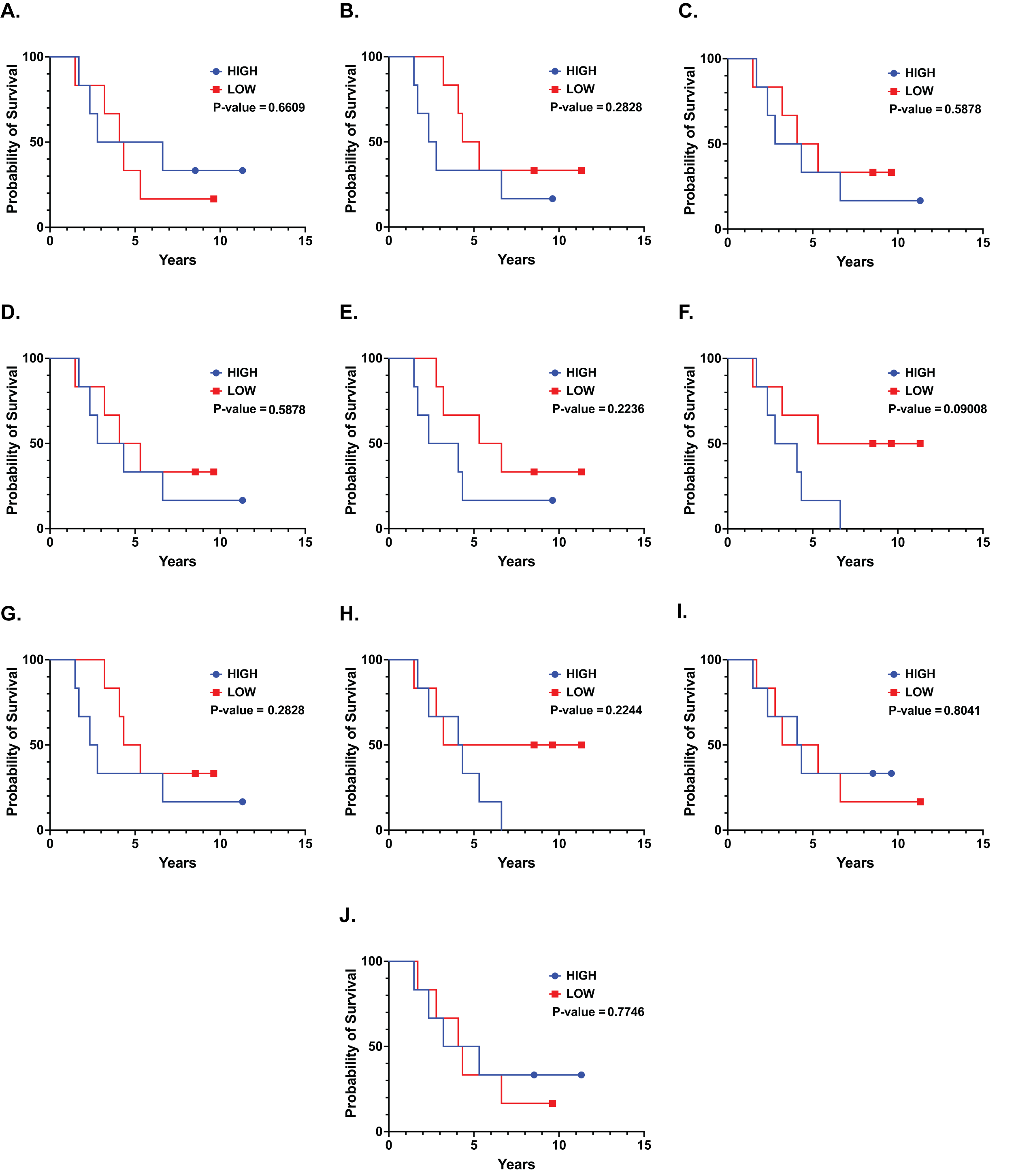

Supplement: Supplementary file 1 — Supplementary Figures and Legends [file 41413_2024_359_MOESM1_ESM.docx]
